# Systematic Review of Genomic‐Based Risk Stratification in Localised Prostate Cancer Treatment Optimisation: Clinical Impact and Health Economic Evidence

**DOI:** 10.1002/cam4.71690

**Published:** 2026-03-09

**Authors:** Juntao Lyu, Fan He, Niall M. Corcoran, Gang Chen, Hadi Akbarzadeh Khorshidi

**Affiliations:** ^1^ Cancer Health Services Research Unit, Centre for Health Policy, Melbourne School of Population and Global Health The University of Melbourne Melbourne VIC Australia; ^2^ Collaborative Centre for Genomic Cancer Medicine The University of Melbourne Melbourne VIC Australia; ^3^ Department of Surgery The University of Melbourne Melbourne VIC Australia; ^4^ Department of Urology Royal Melbourne Hospital Parkville VIC Australia; ^5^ Centre for Health Services Research in Cancer Peter MacCallum Cancer Centre Melbourne VIC Australia

## Abstract

**Background:**

Several genomic risk stratification tests are available to predict the risk of metastasis and mortality for prostate cancer patients at the time of diagnosis. However, the evidence supporting the clinical utility of genomic risk stratification tools is fragmented, posing challenges in assessing their real‐world clinical impact and cost‐effectiveness.

**Objective:**

To review and summarise the clinical impact and health economic evidence of the four types of genomic risk stratification tests and to validate their clinical impact and health economic evaluation outcomes.

**Method:**

A systematic search was conducted in the Scopus, EMB Reviews and Google Scholar databases. Eligible publications were selected based on the eligible patient cohort, genomic test, initial NCCN risk categories, the clinical impact of the genomic test and health evaluation outcomes.

**Conclusion:**

26 clinical impact evidence studies and four health economic evaluation studies were included. Most clinical studies indicated that genomic tests reclassified patients' risk predictions into both lower‐ and higher‐risk groups. The reclassification outcomes influenced patients' treatment decisions between active surveillance and radical treatment. The prognostic value of the genomic tests was validated in terms of biopsy upgrade, metastasis and death. The limited number of health economic studies reported that the Oncotype DX Prostate Score and ProMark were cost‐effective, while the Prolaris was cost‐saving in the US but not in Canada. The evaluation of the Decipher Genomic Classifier at the time of diagnosis was not available. More long‐term clinical evidence is needed, as are updated health economic evaluations, to determine the cost‐effectiveness of integrating genomic risk stratification into prostate cancer treatment decision‐making in clinical practice.

## 1 Introduction

Prostate cancer (PCa) is now the most commonly diagnosed cancer in the developed world [1]. It is predicted that the number of people living with PCa will increase by 68% in the next two decades, and the medical cost increase will be one of the highest of any cancer types [2, 3]. Over 90% of PCa patients present with localised disease, and initial evaluation and subsequent treatment strategies are based on a combination of clinical prognostic factors. These include the Gleason score (tumour grade), prostate‐specific antigen (PSA) level, clinical stage and various prostate biopsy metrics of tumour volume as well as other clinical factors [4, 5]. Alongside a very large number of new annual diagnoses of PCa, the variable and sometimes unpredictable outcomes of the disease make clinical decision‐making challenging. Many cases of PCa follow an indolent course, not requiring treatment with curative‐intent surgery or radiation, whilst others metastasise to distant sites, requiring early and aggressive clinical intervention to avoid or delay death [6]. The two most commonly used PCa risk stratification guidelines currently are the National Comprehensive Cancer Network (NCCN) PCa guideline and the European Association of Urology Guideline (EAU) [7, 8, 9].

The National Comprehensive Cancer Network (NCCN) guidelines have identified clinicopathologic criteria to stratify risk and help guide treatment decisions for individuals with clinically localised PCa [7]. The NCCN initial risk stratification categorises patients into five risk groups: very low‐risk, low‐risk, intermediate‐risk, high‐risk, and very high risk [7]. The intermediate‐risk is further divided into a favourable intermediate‐risk (FIR) and an unfavourable intermediate‐risk (UIR) based on the assessment of patient risk factors, including PSA levels, Gleason scores, and tumour biopsy results. Similarly, the EAU guidelines grade prostate patients into low‐risk, intermediate‐risk (FIR and UIR), and high‐risk groups [10]. The International Society of Urological Pathology Grade Group (ISUP‐GG) provides five ordinal grades based on the Gleason scores [8], which is consistently the single most important prognostic factor and underpins both the NCCN and EAU guidelines. However, these guidelines often do not provide prescriptive or one‐size‐fits‐all advice for the optimal management of clinically localised PCa because the decision‐making process is complex and must be individualised.

According to the recent Australian and New Zealand PCa registry report, over 60% of patients diagnosed with PCa are in low or intermediate‐risk groups [11]. In general, active surveillance is preferred and recommended for low‐risk PCa patients but is only an initial management option for selected patients with intermediate‐risk PCa [12]. Clinicians cannot currently discern clearly at an early stage between PCa with a biologically indolent disposition and cancer with a lethal phenotype (i.e., likely to spread rapidly), as conventional prognostic markers including clinical tumour stage, PSA, and biopsy pathology including Gleason score or ISUP grade group do not predict future tumour behaviour with sufficient accuracy [13]. In recent years, several molecular profiling tests have been developed to address the challenges of overdiagnosis and overtreatment in low‐grade PCa while enhancing the success of personalised therapies for high‐grade and advanced‐stage patients [14].

Recent clinical studies have shown that genomic sequencing analyses of PCa biomarkers can support personalised treatment decisions and reduce uncertainty for patients and healthcare providers [15]. Particularly among the patients with a low‐risk or intermediate‐risk of PCa, genomic tests can provide more assurance for active surveillance with ongoing observation and postponing intervention until definitive treatments are required [16]. The most recent NCCN guidelines also suggested that patients with low or intermediate‐risk PCa with a longer than 10 years of life expectancy could benefit from these genomic risk stratification tests [17].

At the time of this review, four major tissue‐based genomic biomarker tests are commercially available: Decipher Genomic Classifier (GC), Oncotype Dx Genomic Prostate Score (GPS), Prolaris Cell Cycle Progression Test (CCP), and ProMark, from four different companies in the US [5, 18, 19, 20]. Given the significant cost of morbidity, particularly urinary incontinence and erectile dysfunction [13, 21], associated with radical treatments such as external beam radiotherapy (EBRT) or radical prostatectomy (RP), improved risk stratification and prediction through more accurate genomic tests can help avoid unnecessary interventions and better protect patients opting for active surveillance.

Despite the potential advantages of genomic‐based risk stratification for optimising PCa treatment, there is a notable scarcity of health economic studies assessing its cost‐effectiveness. In countries that require formal Health Technology Assessment (HTA) procedures, such as Australia, genomic assays as investigative technologies are subject to assessment of the safety, test accuracy, impact of changes in clinical management, and economic implications of new medical services [22, 23]. Given the increasing integration of genomic testing in precision oncology, a critical appraisal of its predictive accuracy, reclassification impact, and influence on treatment choices is essential to support HTA evaluations and ensure these technologies meet the necessary clinical and economic benchmark for widespread adoption in PCa care.

### 1.1 Review Objectives

This review has two main objectives that serve the purpose of HTA. The first objective is to identify and synthesise the existing clinical evidence on the four PCa genomic tests, focusing on their prognostic accuracy, reclassification metrics compared to the standard‐of‐care initial NCCN risk categories, and the impact on treatment plans among patients with low‐ or intermediate‐risk PCa. Subsequently, the second objective is to review existing health economic evaluation studies and identify evidence and methodological gaps in evaluating genomic‐based risk stratification for optimising PCa treatment decisions.

### 1.2 Research Questions

The research questions to be answered through this systematic review include:

**Q1:** What is the clinical impact of genomic risk stratification compared with the conventional NCCN risk stratification for low or intermediate‐risk PCa patients? More specifically:

**Q1.1:** What are the genomic test reclassification results compared to the initial NCCN risk stratification?

**Q1.2:** What are the clinical impacts of genomic tests regarding aiding treatment decisions?

**Q1.3:** What are the validated prognostic outcomes of these genomic tests' predictive power, such as the likelihood of developing metastasis?

**Q2:** How PCa genomic tests evaluated in health economics? More specifically:

**Q2.1:** What genomic tests have been evaluated, and in what context?

**Q2.2:** What are the health economic modelling strategies?

**Q2.3:** What clinical and cost parameters were used in these evaluations?

**Q2.4:** What are the limitations of existing health economic evidence?

## 2 Materials and Methods

### 2.1 Search Strategy

We used Scopus, which includes the core content of MEDLINE, EMBASE, PubMed, and ScienceDirect, as the primary search engine to search for clinical evidence studies and health economic evaluation studies. This choice was advised by the librarian for its suitability for the research topic, comprehensive coverage, and well‐defined search strategies. We also used the Evidence‐Based Medicine Reviews on Ovid databases, including EBM Reviews—Cochrane Central Register of Controlled Trials (data available until September 2024) to search for clinical evidence studies, and the additional EBM Reviews—Health Technology Assessment (data available until 4th quarter 2016) and EMB Reviews—NHS Economic Evaluation Database (data available until 1st quarter 2016) to search economic evaluation studies. Additionally, we applied the keywords and searched in the Google Scholar database as a complementary step to cross‐check the search results in case of any missing records. We compared the complementary search outcomes with those from other sources to identify additional studies that appear suitable based on their titles, and no key studies were from the complementary search. All the databases were accessed through the University of Melbourne library portal. The literature search was conducted in English on 23 October 2024.

A senior librarian from the Faculty of Medicine, Dentistry and Health Sciences, University of Melbourne, reviewed and reconstructed the search strategies to ensure quality and inclusiveness of searching and reduce errors. The search terms were identified in accordance with the research questions, using broader alternative terms to match the titles, abstracts, and full texts where available in the databases. The search was restricted to human studies, journal articles, and published in English. The search terms and strategy are documented in the appendix document (Appendix 1).

### 2.2 Inclusion and Exclusion Criteria

We used the health economic PICO framework to guide the systematic review, which matched with the suitable (P) patient cohort, the proposed (I) intervention, the (C) comparator, and the analytical (O) outcomes. Based on this framework, we designed a parallel review process for clinical impact evidence studies and health economic studies, respectively. In the clinical impact evidence section, we reviewed available genomic test clinical studies that reported the reclassification outcomes, test accuracy, or the impact on treatment decisions, including any retrospective, prospective, and RCT studies. In the health economic evidence review part, we reviewed the health economic evaluation methods, supporting clinical evidence, costs, and health utilities used in these studies.

- **Population**

The population being analysed is limited to patients initially diagnosed with low or intermediate‐risk PCa based on the NCCN guidelines without genomic testing [7].

- **Intervention**

Genomic‐based risk stratification at the time of diagnosis to guide treatment plans, including both active surveillance and radical treatment plans.

- **Comparator**

The initial risk categories stratified by the NCCN guidelines.

- **Outcomes**

Two sets of outcomes were reviewed in this study: the clinical evidence outcomes and the health economic evaluation outcomes. The clinical evidence outcomes focused on the clinical effectiveness of genomic tests reported from clinical trials or real‐world evidence studies. The outcomes include the validated prognostic outcomes reported from these clinical studies, the net reclassification improvement, and the impact on clinical changes from the genomic test compared to the NCCN risk stratifications. The health economic outcomes focused on evaluating the relative merits of economic measures such as cost‐effectiveness analysis (CEA) or budget impact analysis in the context of genomic testing to guide PCa treatment [24].

The eligible clinical evidence study designs include randomised clinical trials, prospective clinical studies, and retrospective clinical studies, in either clinical trial settings or real‐world data analysis settings.

Studies were excluded if they were:

- Conference proceedings or short reports
- Only reported study outcomes in post‐treatment settings (e.g., post‐prostatectomy).
- Health economic studies that did not report detailed model strategies and evaluation outcomes.

### 2.3 Study Selection

Studies were screened in Covidence by four steps: abstract and title screening, full text screening, quality assessment, and data extraction. Two reviewers (JL and HF) reviewed the title and abstract independently. A third reviewer (HK) then resolved the conflicts between the previous two reviewers. Full text review followed the same procedures with two independent reviewers (JL and HF) and a third reviewer (HK) to resolve the conflicts.

### 2.4 Extract Data From the Included Studies

Data were extracted using a Microsoft Excel spreadsheet with a data extraction table. One reviewer (JL) assessed the selected studies, and two reviewers (JL and HF) then extracted the data from the included studies. The third reviewer (HK) cross‐checked the data table with the original studies to ensure consistency. Data points for clinical evidence studies and health economic evaluation studies are presented in the Table S1.

### 2.5 Quality Assessment and Risk of Bias

The quality of the clinical evidence studies was evaluated using the reporting guideline targeting the Genetic Risk Prediction Studies (GRIPS) [25]. The Grips Statement consists of 25 items to assess the transparency, quality, and completeness of genetic risk‐predicting studies, as well as the limitations, generalisations, and funding declarations to measure the risk of bias. The health economic evaluation studies were assessed using the CHEQUE 2023 checklist with 24 items to evaluate the quality and the transparency of the reporting [26]. JL conducted the quality assessment, and HK examined the outcomes and verified consistency.

## 3 Results

### 3.1 Included Studies

**FIGURE 1:**
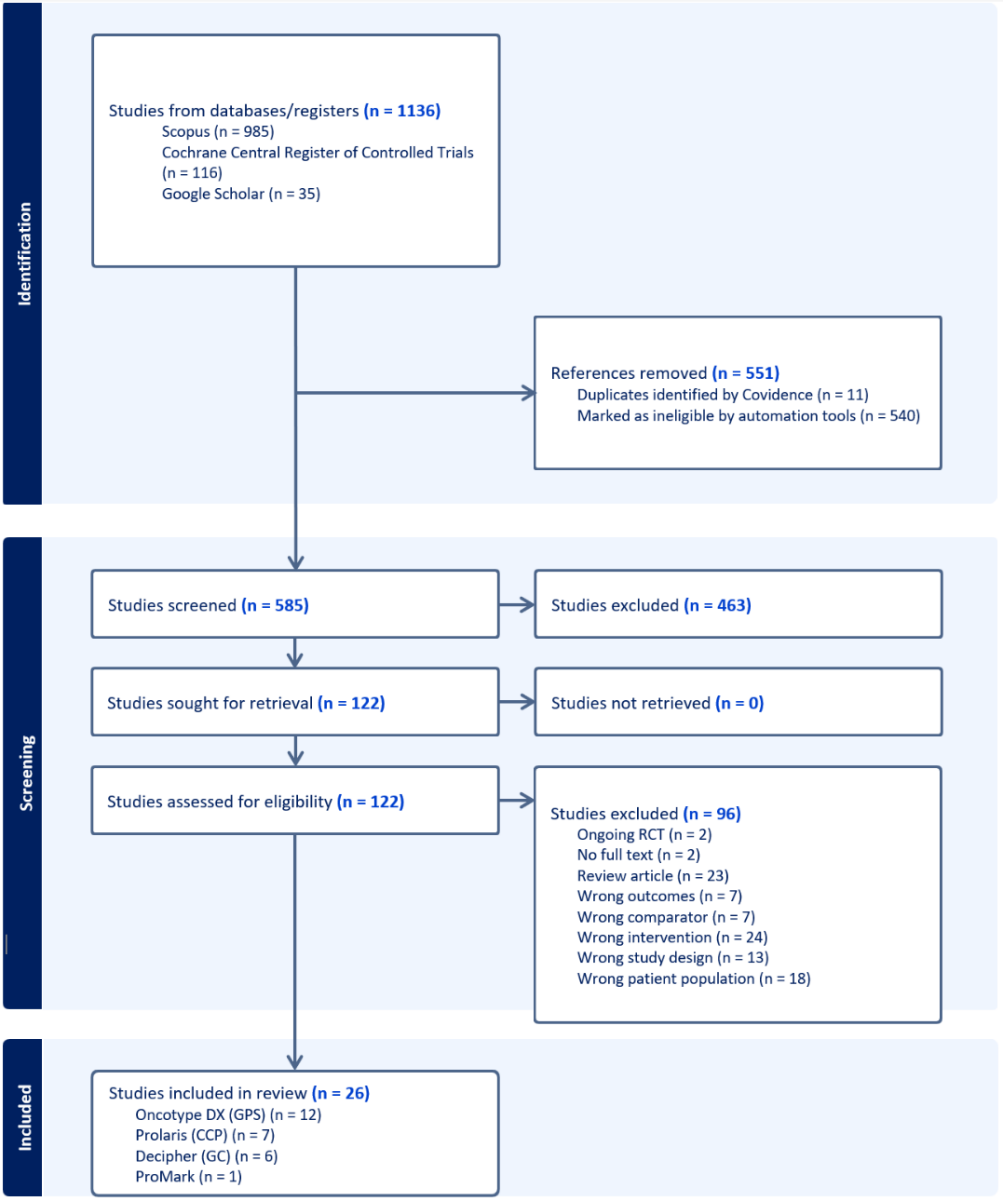
PRISMA flowchart diagram of clinical evidence studies selection process. 26 eligible studies were identified, including 12 Oncotype DX (GPS) studies, 7 Prolaris (CCP) studies, 6 Decipher Genomic Classifier (GC) studies, and 1 Promark study.

Figure 1 shows the PRISMA flow diagrams for screening and selecting clinical evidence studies. 26 clinical evidence studies were selected from the 1136 records, including 12 GPS test studies, seven CCP test studies, six GC test studies, and one ProMark test study.

**FIGURE 2:**
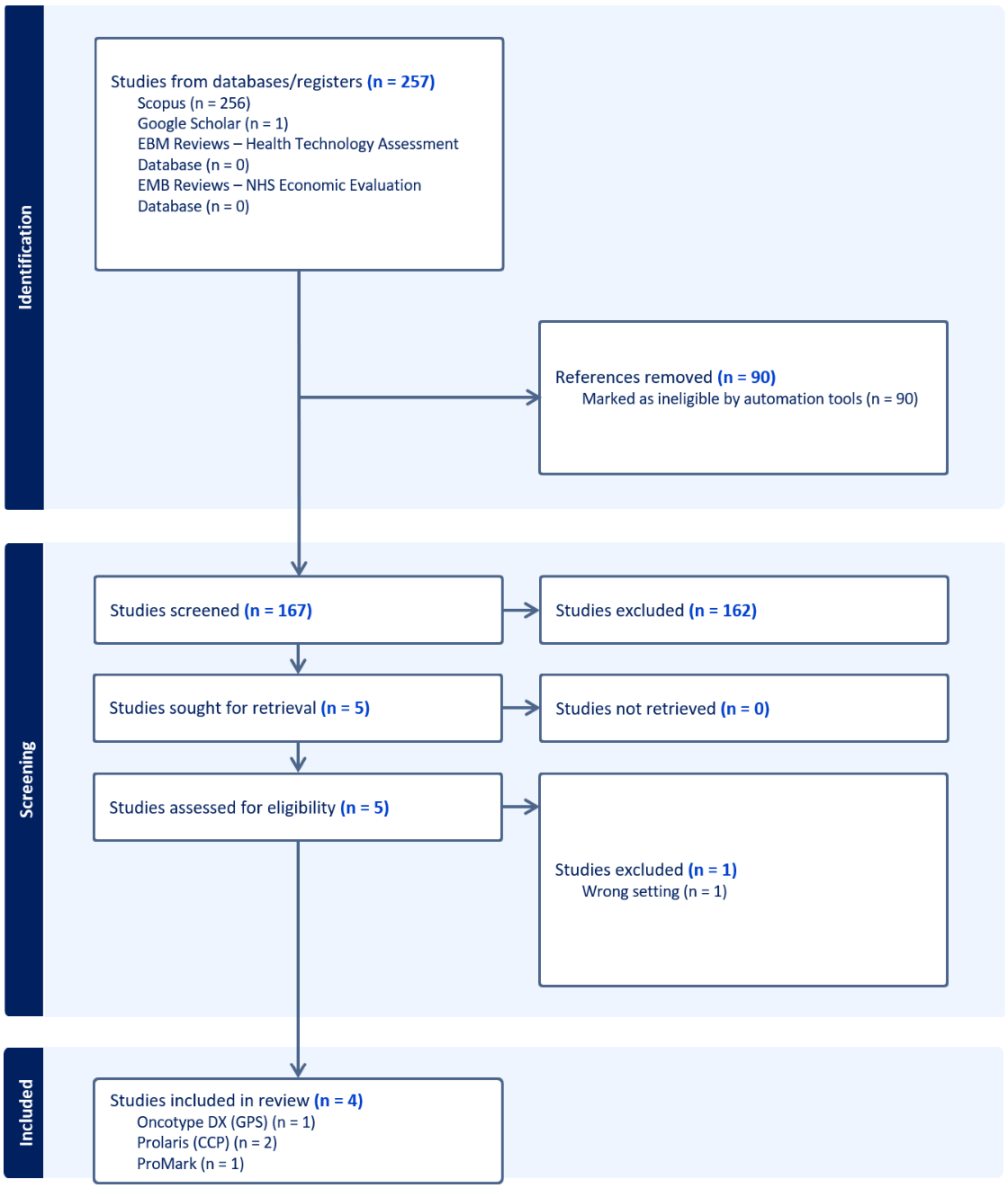
PRISMA flowchart diagram of the health economic studies selection process. Only four eligible health economic studies regarding the four types of genomic risk stratification tools. Decipher Genomic Classifier (GC) has one health economic study focusing on the post‐prostatectomy setting, which is ineligible for this review.

Figure 2 shows the PRISMA flow diagrams for screening and selecting health economic evaluation studies. Four health economic evaluation studies were selected from 257 records, including two CCP studies, one GPS study, and one ProMark study.

### 3.2 Quality Assessment Outcomes

As shown in the Table S2, most of the GRIPS statement items were satisfied (above 75%) except for the missing data handling (23%), supplement information (62%), risk model construction (65%), result validation (65%), model estimates (69%), and model validation (73%). This is because some studies only reported risk reclassification rather than constructing risk predictive models.

Table S3 presents the quality assessment outcomes of the four health economic studies in relation to the CHEQUE 2023 items. Most items were satisfied except for the equity considerations (0%), discount rate (50%), utilities (50%), and software (75%). This is because two of the four studies focus solely on cost.

### 3.3 Clinical Impact of Genomic Tests in Prostate Cancer Treatment Decision‐Making: Evidence Synthesis

We systematically reviewed the clinical evidence of the four genomic tests regarding their risk reclassification outcomes, the impact on treatment decisions, the predictive power validated by patient outcomes, and the contextual factors of these studies.

**FIGURE 3:**
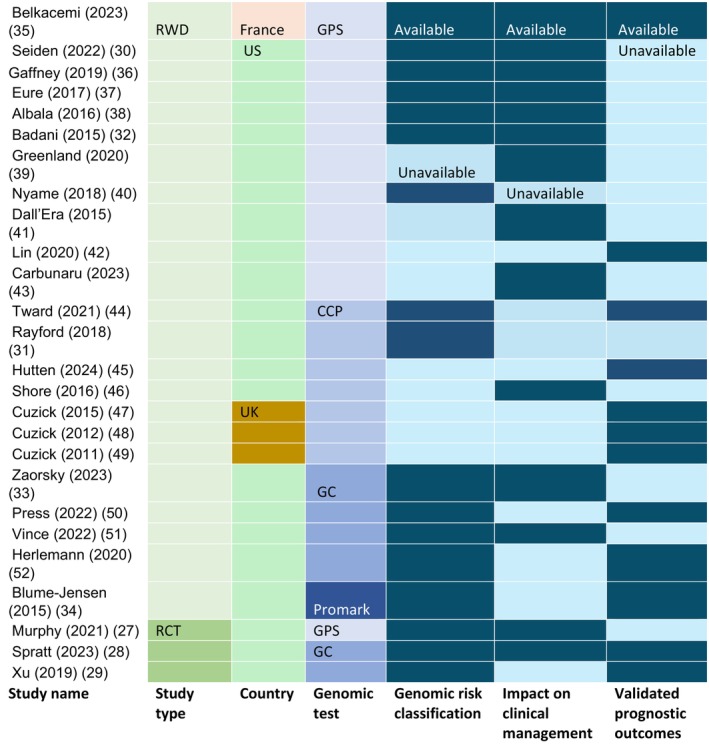
The study feature matrix illustrates the study type and data availability of genomic test clinical studies. Study types, countries, and genomic tests are denoted by distinct colours, with relevant information noted at the first occurrence of each colour. Data availability for each study is indicated by dark blue (available) and light blue (unavailable). Notably, it is uncommon for studies to have data available across all three domains. CCP, Cell Cycle Progression, also called Prolaris; GC, Genomic Classifier, also called Decipher Prostate test; GPS, Genomic Prostate Score, also called Oncotype DX; RCT, randomised clinical trials; RWD, real‐world data.

Figure 3 presents the study feature matrix of these 26 studies, categorised by study type, country, and data availability, regarding genomic risk stratification, impact on clinical management, and predictive power. Most of the included studies are based in the United States (22 out of 26, with two studies also involving Canadian patients), with three studies originating from the United Kingdom and one from France. Most studies used real‐world data (RWD) from registries or hospital records. Only 1/12 clinical studies on the GPS test and 2/6 clinical studies on the GC test were derived from clinical trial data (RCT), although the genomic tests were not their primary research objectives [27, 28, 29]. Most studies reported only one or two of the three main outcomes.

We also summarised more details of each study in the Table S4. The patient age groups vary between 40 and 80 years in each study, with some patient cohorts including only intermediate‐risk or low‐risk PCa patients, and others including both intermediate‐ and low‐risk groups. Most of the studies did not specify the ethnicity of the patient cohorts, except for two studies that reported the African American patient cohort [30, 31]. The follow‐up times vary between a few weeks and over a decade, except that five studies did not specify the follow‐up time [29, 31, 32, 33, 34].

#### 3.3.1 Genomic Risk Stratification Tests and Their Risk Reclassification Outcomes

In clinical practices, the first clinical impact faced by patients and clinicians is the differences between the genomic risk stratification outcomes compared with the initial NCCN risk categories. In the Table S4, we summarised the overall genomic risk reclassification outcomes, which report the overall percentage and number of patients being reclassified into higher or lower risk groups by the genomic test, the impact of the genomic test on treatment decisions, and the validated predictive power of the genomic test. In addition to the overall risk reclassification outcomes, we also summarised the breakdown risk reclassification outcomes (Table S5) according to their initial NCCN risk groups from the intermediate‐risk group (FIR and UIR were not reported), FIR, UIR, and the low‐risk group.

##### 3.3.1.1 Oncotype DX Genomic Prostate Score (GPS)

The GPS assay is a 17‐gene tissue‐based genomic test designed to assess the aggressiveness of very low, low, and favourable intermediate‐risk PCa, aiding in treatment decisions between active surveillance and curative intervention, with additional use in intermediate‐ and high‐risk cases for treatment escalation or de‐escalation [5]. The GPS outcomes range from 0 to 100, with higher scores indicating an increased risk of developing aggressive disease [5].

We identified one RCT study from Murphy (2021), and the study reported that the GPS reclassified 14.3% of initial NCCN FIR patients to the low‐risk group and 21.4% to the high‐risk group [27]. For the initial NCCN low‐risk PCa patients, the GPS reclassified 29.4% into the intermediate‐risk, and none of them were reclassified as high‐risk [27]. We identified 11 RWD studies reporting the reclassification results of the GPS test. Three RWD studies reported the reclassification outcomes of the intermediate‐risk group, including 11.1%, 20.7% and 10.1% to the low‐risk group, and 2.7%, 1.7% and 0% to the high‐risk group, respectively [35, 36, 37]. Four RWD studies reported the GPS reclassification outcomes of the FIR group, and one study did not find any changes between the initial NCCN risk categories and the genomic risk stratification outcomes [30, 32, 38, 39]. The GPS also reclassified 16.7% of the UIR patients to the FIR group and 33.3% to the high‐risk group [38].

Seven RWD studies reported the GPS reclassification results among the patients with low‐risk PCa, and none of them were reclassified into the high‐risk group [30, 32, 35, 36, 37, 39, 40]. However, as summarised in Table 2, some of the low‐risk PCa patients were reclassified into the intermediate‐risk group by the GPS test, and the range varies from 4.2% to 35.7% across different studies [30, 32, 35, 36, 37, 39, 40].

##### 3.3.1.2 Decipher Prostate Genomic Classifier (GC)

The Decipher Genomic Classifier (GC) measures the expression of 22 RNA biomarkers and provides a continuous scale (0 to 1) to indicate the risk of clinical metastasis and cancer‐related mortality [41]. There were six studies identified where the GC test was used as a risk stratification tool to guide PCa treatment plans, including analytical outcomes from two clinical trial settings and four real‐world data retrospective cohort studies [28, 29, 33, 42, 43, 44].

The most recent completed randomised clinical trial is the Radiation Therapy in Treating Patients With Stage II PCa (NCT00033631) [45]. Spratt (2023) reported that the GC test reclassified 81% of the intermediate‐risk patients to the low‐risk group and 9% to the high‐risk group [28]. Another completed clinical trial is the Evaluation of Gallium‐68‐HBED‐CC‐PSMA Imaging in PCa Patients (NCT02611882), which also examined the GC risk stratification against the initial NCCN risk levels [46]. Xu (2019) reported that 69.6% of the NCCN intermediate‐risk PCa patients were reclassified into the low‐risk group and 30.4% to the high‐risk group [29].

The most recent RWD study is derived from the SEER (Surveillance, Epidemiology, and End Results) program registries in the US [33]. The reclassification results by the GC test show that 42% were reclassified from the FIR group to the low‐risk group and 28.5% to the high‐risk group; 33.4% were reclassified from the UIR group to the low‐risk group, and 41.3% were reclassified from UIR to the high‐risk group [33]. For the 158 intermediate‐risk PCa patients, 41.5% were reclassified into the low‐risk group, and 24.7% were reclassified into the high‐risk group [33]. Similarly, the low‐risk group was also reclassified by the GC test as 28.5% intermediate‐risk and 17.2% high‐risk [33].

The other two groups of FIR patients were reported based on the Genomic Resource Information Database in the US [43, 44]. Vince (2022) found that GC reclassified 22% FIR into the low‐risk group and 4% FIR into the high‐risk group, while Herlemann (2020) found that the GC test reclassified 79.1% FIR into the low‐risk group and 7.7% FIR into the high‐risk group [43, 44]. Vince (2022) also found that 4.5% low‐risk PCa patients were reclassified by the GC test as high‐risk [43]. Press (2022) also reported the GC reclassification results from 133 patients with biopsy Gleason Grade one and two without breaking down the initial NCCN risk groups, and 10.3% of them were reclassified as high‐risk by the GC test [42].

##### 3.3.1.3 Prolaris Cell‐Cycle Progression Score (CCP)

Prolaris Cell‐Cycle Progression Score (CCP) is an RNA 46‐gene assay that utilises quantitative reverse transcription polymerase chain reaction (RT‐PCR) to measure the expression of 31 cell cycle progression genes and 15 housekeeping genes [47]. It generates a CCP score (0–10), with higher scores indicating a greater likelihood of disease progression, metastasis and biochemical recurrence [47].

We identified seven eligible RWD clinical studies and no eligible RCT studies. Three of them were UK‐based studies reported by the same first author, Cuzick, in different years (2011, 2012, 2015) [48, 49, 50]. However, these three studies focused on validating the CCP's predictive power rather than the reclassification metrics in clinical settings.

Two RWD studies reported the reclassification metrics by the CCP. Tward (2021) reported the reclassification outcomes derived from the USA's five tertiary PCa centres [51]. For patients with initial NCCN FIR PCa, 2% were reclassified into the low‐risk group, and none were reclassified into the high‐risk group. The UIR group had 73% reclassified into the low‐risk group, and none were reclassified into the high‐risk group. The CCP also reclassified 27% (52/193) from the high‐risk to the low‐risk group [51].

Rayford (2018) reported the data derived from a community urologic oncology practice in Memphis (USA), consisting of 150 African American and 60 Caucasian men [31]. The CCP reclassified the NCCN intermediate‐risk group with 32.9% to the low‐risk group and 17.6% to the high‐risk group. The CCP also reclassified the NCCN low‐risk group with 20% to the intermediate‐risk group and 1.8% to the low‐risk group [31].

##### 3.3.1.4 ProMark Prostate Cancer Genomic Test

The ProMark genomic test is a protein‐based biomarker assay that quantifies eight proteins involved in cell signalling, stress response and cell proliferation using quantitative multiplex proteomics imaging (QMPI) on prostate biopsy tissue [52]. The test generates a ProMark Score (1 to 100), which predicts the likelihood of high‐risk disease, non‐organ‐confined cancer, or Gleason Group ≥ 3 post‐radical prostatectomy.

One Canada‐based RWD comparative study reported using ProMark as risk stratification after diagnosis. Blume‐Jensen (2015) validated the clinical utility of ProMark from a study cohort derived from a single hospital in Canada [34]. ProMark test reclassified 88 NCCN intermediate‐risk PCa patients into 13.6% low‐risk and 29.5% high‐risk. It also reclassified 94 NCCN low‐risk PCa patients into 62.8% intermediate‐risk and 8.5% high‐risk [34].

##### 3.3.1.5 Genomic Test Risk Reclassification: Pooled Outcomes

**FIGURE 4:**
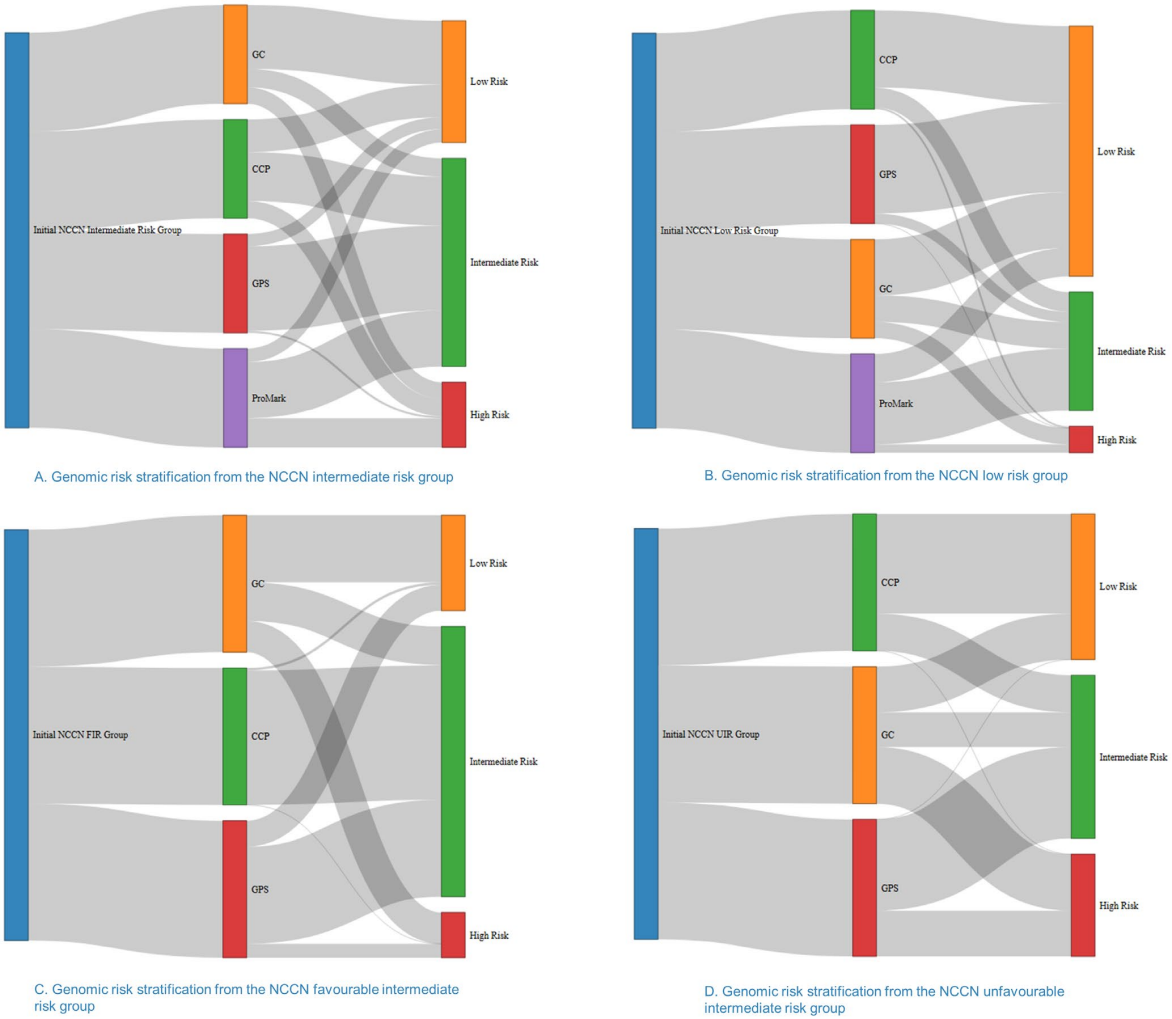
The Sankey diagram illustrates how the four genomic test tools reclassified prostate cancer patients from their initial NCCN risk levels to different new genomic‐based risk levels. Genomic risk stratification tools significantly shifted the initial NCCN risk levels. CCP, cell cycle progression, also called Prolaris; FIR, favourable intermediate‐risk; GC, Decipher Genomic Classifier, also called Decipher Prostate test; GPS, Genomic Prostate Score, also called Oncotype DX; NCCN, National Comprehensive Cancer Network; UIR, unfavourable intermediate‐risk.

Based on the initial NCCN risk groups, we pooled the genomic risk reclassification outcomes for each genomic test, as illustrated by Figure 4. We found significant discrepancies in risk reclassification among the four genomic tests. For intermediate‐risk PCa patient groups, the GC reclassified 64.6% of them as low‐risk and 16.7% of them as high‐risk [28, 29, 33]; the CCP test reclassified 32.9% of them as low‐risk and 17.6% of them as high‐risk [31]; the ProMark test reclassified 13.6% of them as low‐risk and 29.5% of them as high‐risk [34]; the GPS reclassified 12.2% of them as low‐risk and 2.1% of them as high‐risk [35, 36, 37]. For the FIR PCa patient groups, the GC reclassified 48.9% of them as low‐risk and 22.9% of them as high‐risk [33, 43, 44]; the GPS test reclassified 19% of them as low‐risk and 10.1% of them as high‐risk [27, 30, 32, 38, 39]; the CCP test reclassified 1.8% of them as low‐risk and none as high‐risk [53]; and the ProMark test does not have data in this group. For the UIR PCa patient groups, the CCP reclassified 72.9% of them as low‐risk and none as high‐risk [53]; the GC test reclassified 33.4% of them as low‐risk and 41.3% of them as high‐risk [33]; the GPS test reclassified 16.7% of them as FIR and 33.3% of them as high‐risk [38]; and the ProMark test does not have data in this group. For the low‐risk PCa patient groups, the ProMark reclassified 62.8% of them as intermediate‐risk and 8.5% of them as high‐risk [34]; the GC test reclassified 26.85 of them as intermediate‐risk and 16.4% of them as high‐risk [33, 43]; the CCP test reclassified 20% of them as intermediate‐risk and 1.8% as high‐risk [31]; and the GPS reclassified 10.3% of them as intermediate‐risk and none as high‐risk [27, 30, 32, 35, 36, 37, 39, 40].

#### 3.3.2 Impact on Treatment Decisions and the Predictive Power of the Genomic Risk Stratification Test

In this section, we summarised the studies that reported either the change of treatment plans after the genomic test or the validation of patient outcomes compared to the genomic test risk predictions.

##### 3.3.2.1 GPS

As presented in Table 1, we identified nine GPS studies that reported the impact on treatment decisions after the genomic risk reclassification, and only three reported the validation outcomes for the GPS test's predictive power [27, 30, 32, 35, 36, 38, 39, 54, 55]. Five GPS studies reported an increase in AS utilisation after the GPS test, and these studies did not report the predictive power and the associated patient outcomes [32, 35, 36, 39, 55]. Three studies reported an increase in radical treatment after the GPS test, without predictive power and the patient outcomes [27, 30, 54]. One GPS study reported the treatment intensification after the GPS test and found a significant correlation between GPS and the percentage of Gleason Grade 4 or higher patterns in surgical samples [38]. The other two GPS studies validated the predictive power of the GPS test. Greenland (2020) found that the increase in the GPS test was associated with moderate or severe stromal reaction (*p* < 0.001) and chronic inflammation (*p* < 0.001) [37]. However, Lin (2020) found that there was no association observed between GPS and subsequent biopsy upgrade (*p* = 0.48) [56].

**TABLE 1:** Summary of the health economic studies.

	Gustavsen (2020)	Bauman (2017)	Chang (2019)	Roth (2015)
Assessed test	CCP test	CCP test	GPS	ProMark
Country	US	Canada	US	US
Patient cohort	Low or intermediate‐risk PCa patients	Low or intermediate PCa patients in Ontario	65‐year‐old men with very low, low, or FIR	60‐year‐old men with biopsy Gleason 3 + 3 or 3 + 4 PCa
Comparator	Standard of care without CCP	Standard of care without CCP	Standard of care without GPS	Standard of care without ProMark
Type of economic evaluation	Cost‐saving analysis	Budget impact analysis	Cost‐effectiveness analysis	Cost‐effectiveness analysis
Time horizon	10 years	5 years	20 years	Lifetime
Discount rate	No discount	No discount	3% per year	3% per year
Type of model	Deterministic Markov model with 1‐year interval	Deterministic budget impact model	Markov state‐transition model with 1‐year interval	Markov state‐transition model with 1‐month interval
Perspective	Commercial health insurance payers	Public healthcare payers	Payer	Payer
Willingness to pay (WTP) thresholds	No WTP	No WTP	$100,000 per QALY	Ranging from $10,000 to $150,000 per QALY
Primary outcomes	(1) CCP increased AS rates. (2) CCP saved $2129 per patient in 10 years	(1) CCP increased AS rates. (2) CCP saved $8 million with spending an extra $47.9 million on tests	(1) GPS increased the QALYs by 0.11. (2) GPS cost an extra $3522 per patient. (3) ICER = $31,394/QALY	(1) ProMark increased the QALYs by 0.04. (2) ProMark saved costs by $700 per patient.
Conclusion by the authors	CCP is cost‐saving but limited to low and intermediate‐risk patients	Savings made by the CCP test are too small to offset the cost of the test.	GPS is cost‐effective with minimal dependence on choice and cost of treatment modality or AS protocol	ProMark is expected to dominate against the standard of care practices across various plausible assumptions

*Note:* The Prolaris (Cell Cycle Progression, CCP) test was estimated to be cost‐saving in the United States setting; however, this cost‐saving effect was not observed in the Canadian context. In the United States, both the Oncotype DX (Genomic Prostate Score, GPS) and Promark tests were estimated to be cost‐effective.

Abbreviations: ICER, incremental cost effectiveness ratio; QALY, quality adjusted life years.

##### 3.3.2.2 GC

Three GC studies reported the impact on treatment decisions after reclassification, while four GC studies reported the validated predictive power, with only one study linking both the reclassification outcomes and the impact on treatment decisions [28, 33, 43]. One GC study reported an increase in AS utilisation after the GC test, without the validation of the predictive power [33]. One GC study reported an increase in radical treatment after the GC test informed of the higher risks, and no validation of the predictive power was reported [43]. Another GC study (RCT) reported the treatment intensification after the GC test, and the GC score was validated as a significant predictor for disease progression, metastasis and PCa‐specific mortality [28]. The other two GC studies also found that the GC score was significantly associated with biopsy upgrade and adverse pathology [42, 44].

##### 3.3.2.3 CCP

One CCP study reported the impact of treatment without clear genomic reclassification outcomes and found that the CCP test caused a change in actual treatment in 47.8% of patients [57]. 24.2% changed from non‐interventional to interventional after the CCP test, and 14.2% changed from non‐interventional to interventional after the CCP test [57]. The other five studies reported the validated predictive power for the CCP test and found that the CCP score is a significant predictor for metastasis and PCa‐specific mortality [48, 49, 50, 51, 58].

##### 3.3.2.4 ProMark

Only one ProMark study reported the genomic risk reclassification outcomes and the predictive power, but no data on the impact of treatment decisions [34]. The study examined the predictive power of the ProMark test in identifying the favourable pathology (Surgical Gleason ≤ 3 + 4 and organ‐confined disease) and non‐favourable pathology features (Surgical Gleason ≥ 4 + 3 or non‐organ‐confined disease) [34]. The predictive power to distinguish favourable from non‐favourable pathology is AUC = 0.68, *p* < 0.0001, and OR = 20.9, indicating that patients with a high ProMark score are 20.9 times more likely to have non‐favourable pathology compared to those with a low score [34].

### 3.4 Health Economic Evaluations

#### 3.4.1 Evaluation Methods and Outcomes

Among the final four eligible health economic studies ranging from 2015 to 2020, two of them evaluated the cost savings of using the CCP test for PCa treatment decision‐making in Canada and the US [59, 60]. The other two evaluated the cost‐effectiveness of using the ProMark test and the GPS in the US [61, 62]. No eligible studies were found to assess the use of GC to guide PCa treatment decision‐making.

As summarised in Table 1, Bauman (2017) completed an HTA assessment of the Prolaris test, which conducted a 5‐year budget impact analysis of using the CCP test in Ontario, Canada, with an assumed patient cohort with newly diagnosed low or intermediate localised PCa [59]. The study estimated that the CCP test was not cost‐saving because the cost of the test (USD 47.9 million) was much higher than the savings it made by more patients choosing active surveillance (USD 7.3 million) in Ontario, Canada [59].

However, Gustavsen (2020) did another 10‐year cost‐saving analysis of CCP test in the USA and found that taking the CCP test could save USD 2129 per patient because of the increased use of active surveillance [60].

The other two health economic studies evaluated the health outcomes and the economic outcomes of the two genomic test products compared with the NCCN standard of care practices. Roth, et al. (2015) assessed the use of the ProMark test on a simulated patient cohort, who are aged 60 years with biopsy Gleason 3 + 3 or 3 + 4 PCa in the USA. The study employed a Markov state‐transition model with a 1‐month interval to estimate the disease progression of six health states, including active surveillance, EBRT, brachytherapy, RP, recurrence and death. Ultimately, this study compared the scenarios of patients reclassified by the genomic test with patients not genomically tested, and estimated that using the ProMark test could increase 0.04 QALYs per patient while saving USD 700 per patient over the lifetime of the simulated cohort [61]. Chang, et al. (2019) evaluated the cost‐effectiveness of using the GPS test on a patient cohort of 65 years old with low or FIR against the standard of care practice. The study employed a Markov state‐transitioning model with a 1‐year interval for 20 years and estimated that the GPS could increase 0.11 QALYs per patient with the ICER of USD 31,394 per QALY [62]. Both studies concluded that genomic tests were cost‐effective and sometimes dominant [61, 62]. However, both studies acknowledged that there was no direct clinical evidence to support the treatment effectiveness, which directly impacts the projection of health outcomes [61, 62].

#### 3.4.2 Assessed Clinical Evidence

**FIGURE 5:**
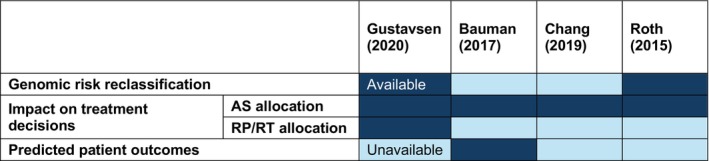
Clinical evidence data availability is denoted by “available” (dark blue) and “unavailable” (light blue) for each study. Whilst all studies utilised active surveillance (AS) allocation evidence in their evaluations, only two studies employed genomic risk reclassification evidence. Furthermore, only one study incorporated genomic‐based treatment allocation evidence, and similarly, only one study utilised genomic‐based patient outcome evidence. Notably, no health economic studies employed clinical evidence from all four domains concurrently. RP, radical prostatectomy; RT, radiotherapy.

The clinical evidence of effectiveness serves as the foundation for conducting health economic evaluations. We summarised the clinical evidence used in the four health economic studies and found that only a small number of clinical evidence studies were used. The evidence matrix (Figure 5) presents the types of clinical evidence used in each study. All health economic evaluation studies focused on the clinical evidence of AS allocation, which is the key to driving the different clinical and economic outcomes [59, 60, 61, 62]. Only two studies used the clinical evidence of genomic risk reclassification [60, 61]. Regarding the treatment distribution across different types of treatment, one study used the direct clinical evidence [59]. Another study used the assumed treatment pathways from a different type of genomic test [61], and two studies used the same standard of care treatment pathways for the genomic test arm [60, 62]. Regarding the predicted disease progression outcomes, only one study used the clinical evidence directly from the genomic test validation study, focusing on the low‐risk PCa patients who elected AS [60]. All studies assumed the same disease progression features for both the genomic test group and the standard of care group due to insufficient clinical evidence [59, 60, 61, 62]. The limited clinical evidence incorporated into these health economic evaluation studies may be attributed to the unavailability of relevant clinical data at the time these analyses were conducted. Since then, however, more recent and comprehensive clinical evidence has become available for use in future evaluations.

#### 3.4.3 Assessed Costs and Utilities

Another challenge in assessing the role of genomic tests in guiding PCa treatment is determining what cost components should be included and what health utilities are available to use. We identified the common cost items used across the four studies, including the costs of diagnostics or tests, active surveillance and radical treatment. We also identified the associated health utilities. However, different components were used across studies, reflecting the variety of treatment pathways for PCa patients. Table 2 shows the cost components and the associated health utilities assessed in the four studies, with the absence of evidence highlighted in red. We summarised the cost items and calculating methods by either the cost per unit or the average cost per year. The currency was converted to USD if it was not reported so in each study. All four studies reported the genomic test cost [59, 60, 61, 62], two studies reported the cost of the PSA test [59, 62], and only one study listed the cost of urologist visits, counselling and biopsy [59]. Three studies assessed the cost of AS per year [59, 60, 62], and one study evaluated AS per month [61]. In the treatment module, all studies assessed the cost of prostatectomy and radiation therapy [59, 60, 61, 62]; two studies considered the cost of brachytherapy [61, 62]; two studies considered the cost of ADT [60, 62]; and only one study considered the cost of adjuvant radiation therapy and castrate‐resistant PCa treatment [60]. In terms of the post‐treatment (and treatment complications), three studies considered the cost of recurrence [59, 61, 62]; three studies considered the cost of post‐RT and post‐RP care [59, 60, 62]; and only one study considered the detailed cost of treating other complications such as erectile dysfunction, urinary incontinence, radiation toxicity and other surgical complications [61]. We also found that Roth (2015) and Chang (2019) extracted very different health utility values from various studies [61, 62, 63, 64].

**TABLE 2:** Summary of the costing components and health utilities.

Components	Gustavsen (2020)	Bauman (2017)	Chang (2019)		Roth (2015)	
	Cost (USD)	Cost (USD)	Cost (USD)	Health utility	Cost (USD)	Health utility
Diagnostics						
Genomic test	$3400	$3400	$3161		$3800	
PSA test		$23				
Urologist visit		$20				
Counselling		$48				
Biopsy		$1501				
AS						
Active surveillance	$1066 per year	$1019 first year; $352 per year afterward	$2657 per year	0.99	$840 per year	0.83
Treatment						
Prostatectomy	$10,604 first year	$14,551 per year	$9562 first year		$8547 first year	0.8
Rachytherapy			$15,781 first year		$14,106 first year	0.8
Radiation therapy	$34,116 first year	$12,049 per year	$30,299 first year		$27,084 first year	0.8
Androgen deprivation therapy	$2933 per year		$2870 first year; $2004 per year afterwards			
Adjuvant salvage radiation therapy	$29,101 first year					
Castrate‐resistant PCa	$122,323 per year					
Death						
PCa death			$45,652	0	$40,807	0
Other causes of death				0	$5000	0
Treatment complications						
Recurrence		$5972 per year	$18,720 first year; $2475 per year afterwards	0.45	$1776 per year	0.68
Biochemical failure				0.78		
Post‐RT care	$845 per year	$4842 per year	$1033 first year; $533 per year afterwards	0.92		0.8
Post‐RP care	$774 per year	$2278 per year	$3664 first year; $533 per year afterwards	0.92		0.8
Erectile dysfunction treatment					$156 per year	0.73
Urinary incontinence treatment					$504 per year	0.63
Other surgical complications treatment					$8256 per year	
GU radiation therapy toxicity treatment					$216 pr year	0.68
GI radiation therapy toxicity treatment					$1560 per year	0.76

*Note:* All costs are reported as US dollars in the year of the study. Four health economic studies employed markedly different health states, events, costing methods and health utility values, despite three of them originating from the same country.

Abbreviations: AS, active surveillance; GI, gastrointestinal radiation injury; GU, genitourinary; PCa, prostate cancer; PSA, prostate‐specific antigen.

## 4 Discussion

### 4.1 Clinical Impact Evidence

We systematically reviewed 24 clinical impact evidence studies and evaluated four types of genomic risk stratification tests to guide PCa treatment decision‐making at the time of diagnosis. Most studies reported results regarding risk reclassification; however, only a limited number provided evidence on how the genomic risk stratification affected the treatment decisions and the predictive power regarding disease outcomes. We found several other review studies that assessed a wide range of genomic tissue biomarkers for AS candidate selection by their prognostic accuracy values, but none of them reported the risk reclassification outcomes at the time of diagnosis [5, 65, 66, 67, 68]. To the best of our knowledge, this review is the first to systematically summarise the comparative and quantified risk reclassification metrics for the four types of genomic risk stratification tests, and it is also the first review to examine the impact of genomic risk reclassification outcomes in clinical practice.

#### 4.1.1 Impact on Risk Levels

When comparing the risk reclassification outcomes reported in individual studies, two of the GC test studies reported the highest proportion of reclassifying patients from intermediate‐risk and FIR to low‐risk (81% and 79.1%, respectively) [28, 44]. One of the CCP test studies reported the highest proportion of reclassifying patients from UIR to low‐risk (72.9%) [53]. In terms of reclassifying patients from intermediate‐risk groups to high‐risk groups, two of the GC test studies reported the highest proportions of reclassifications from intermediate‐risk and UIR to high‐risk (30.4% and 41.3%, respectively) [29, 33]; one of the GPS test studies reported the highest proportion (38.9%) of reclassification from FIR to high‐risk [38]. Regarding the reclassifications in low‐risk groups, one ProMark study reported the highest proportion of reclassification from low‐risk to intermediate‐risk (62.8%) [34]; one of the GS test studies reported the highest proportion (17.2%) of reclassification from low‐risk to high‐risk [33].

#### 4.1.2 Impact on Active Surveillance Uptake

Recognising the suitability of undertaking AS is one of the main clinical goals of genomic risk stratification [52]. However, very few studies have reported the impact on clinical management, especially regarding the uptake of AS. Many studies utilised retrospective real‐world data to re‐examine the association between genomic testing and clinical management strategies and often failed to clarify whether the genomic test was conducted before or after treatment decisions were made [33, 38, 43]. This ambiguity introduces potential confounding factors and limits the ability to establish a clear causal relationship between the genomic test and AS uptake. Most clinical evidence studies have utilised retrospective patient data from registries or hospital records, which often exhibit diverse patient characteristics. These various channels of data sources make it difficult to compare the outcomes across the four genomic tests directly. We also need to be aware that genomic risk reclassification outcomes are highly dependent on the disease characteristics of the patient cohort. For example, Rayford (2018) found that the CCP test reclassified significantly more African American patients to higher risk groups than their Caucasian peers from their initial NCCN risk categories (24% vs. 10%) [31].

Additionally, genomic test results and the impact on treatment decisions were also evaluated in conjunction with several other factors in these studies. For instance, despite higher genomic risk scores found among the African American patients, the treatment decisions were often influenced more by factors such as health literacy, family history of PCa and health insurance status [27, 29]. These patient characteristics, including age, ethnicity, baseline Gleason score, PSA and other relevant clinical factors, should be carefully considered when evaluating the clinical utility of genomic risk stratification in guiding PCa treatment decisions.

Despite those limitations, we could still identify that a genomic low‐risk outcome, regardless of the types of genomic tests, increased the uptake of AS among PCa patients with intermediate or low NCCN risk levels [32, 33, 35, 36, 39, 69].

#### 4.1.3 The Validated Prognostic Value of Genomic Risk Stratification

Comparing the initial genomic risk assessment results with disease progression outcomes to validate the predictive power is difficult due to multiple reasons, especially the limited longitudinal data and the variable treatment regimens patients often receive [41]. Nevertheless, the GPS test studies reported both significant and insignificant associations between the test outcomes and the biopsy upgrade; its independent prognostic utility remains uncertain and may be more informative when combined with traditional clinical and histopathological factors [37, 38, 56]. The GC test studies reported consistent significant prognostic values in terms of predicting biopsy upgrade, metastatic disease and PCa‐specific mortality [28, 29, 42, 44]. The CCP test studies reported significant prognostic outcomes regarding death and metastasis, especially for 3–10 years of prediction [48, 49, 50, 51, 53, 58]. The ProMark study only reported the significant prognostic value regarding the ability to identify the favourable and non‐favourable pathology for PCa patients [34].

### 4.2 Health Economic Evidence Discussion

We systematically reviewed four health economic evaluation studies regarding the GPS, CCP and ProMark genomic tests; no studies were found to evaluate the use of the GC risk stratification to guide treatment decisions before RP. This is because the GC test was initially developed for post‐prostatectomy risk stratification and later expanded to guide risk stratification at the time of diagnosis [70]. A recently published systematic review looked into the cost‐effectiveness of all genomic medicine in cancer control, including PCa screening technologies such as the Sotckholm3 test and the Prompt Prostate Genetic Score, and metastasis management technologies such as Olaparib [71]. However, this review neglected to mention the health economic studies of the PCa genomic risk stratification tests [71]. Another study reviewed eight health economic studies on seven types of PCa genomic risk stratification tests, including the four health economic studies in this review [72]. However, that study mainly focused on the health economic evaluation outcomes and did not specifically assess the clinical evidence, cost components and quality of life evidence metrics used in these studies [72].

We assessed the four health economic studies and found that they have very limited clinical evidence to support their conclusions (Figure 5), particularly regarding the treatment effects after patients were reclassified into different risk groups [59, 60, 61, 62]. We compared the clinical evidence used in these four health economic studies with our clinical evidence review and found that only a small number of clinical evidence studies were used, and many new clinical studies have become available since these health economic studies were published [59, 60, 61, 62]. We noticed that the cost parameters vary greatly between each study, and the two cost‐effectiveness studies also used very different health utilities from the baseline values to the treatment and complications derived from various sources [61, 62]. A recent longitudinal RCT study has provided more comprehensive and consistent health utility values for PCa treatment and health states, which might be helpful to mitigate the differences in PCa health utility values [73].

All current health economic studies of genomic risk stratification employed a deterministic Markov transition model relying on inconsistent health states, cost components, utility values and fixed transition probabilities. The economic model did not accurately capture prostate cancer disease progression features, particularly in terms of time‐dependent transitions and the heterogeneity in patients' trajectories over time across the health states of AS, definitive treatment, and post‐treatment management, and the possibilities of skipping treatment or metastasis [59, 60, 61, 62]. Subsequently, the current four health economic studies have very differently defined disease events and relevant costs, which caused difficulties in comparing the economic evaluation outcomes across studies [59, 60, 61, 62]. To address the complexities of time‐dependent transitions and heterogeneity in patient trajectories, future studies should consider employing more sophisticated modelling techniques, such as microsimulation and discrete event simulation, to capture the diverse scenarios of PCa patient journeys.

## 5 Limitations

This study has two major limitations. First, this review is limited to the four types of commercially available tissue biomarker genomic risk stratification tests in PCa treatment decision making. There are many other types of genomic tests used in PCa diagnosis and risk stratification that have been purposely excluded from this review. For example, the PTEN/TMPRSS2:ERG assay is another type of tissue biomarker genomic test product developed by Metamark (US); however, it was reported with insufficient clinical evidence and is not recommended in the most recent NCCN guidelines [66]. Other blood or urinary biomarker‐based genomic tests, such as SelectMDx, ConfirmMDx, EPO Prostarix, DNA‐Ploidy, ProstatePx, NADiA ProsVue Slope and PCMT, are also used in diagnosing significant PCa; however, they are either initial tests to inform biopsy or tests used in post‐prostatectomy and have very limited ability to predict the risk of death, metastasis and progression [5, 65, 72].

Second, although we tried to pool the outcomes together from various clinical studies, it is not feasible at this stage to provide statistically robust meta‐analysis outcomes for the risk reclassification metrics, the impact on treatment and the predictive power validated by patient outcomes. We are cautious about various uncontrolled contextual factors, including patient cohort characteristics, follow‐up times, study length and study designs. Considerably, many studies stated that genomic testing was not the original primary research objective in their data sources; selection bias that potentially favours patients with more severe symptoms may have influenced outcomes [28, 29, 37, 44]. The assessment of predictive power is further complicated by the limited data of long‐term follow‐up studies and the heterogeneity of endpoints, which range from metastasis and mortality to biopsy upgrade and adverse pathological outcomes. These variations in study design, patient selection and outcome measures present significant challenges in synthesising evidence and drawing robust conclusions about the clinical utility of genomic testing in management strategies. To mitigate this, we first provided a detailed summary of the clinical impact evidence for each study in the Table S4, acknowledging the variability and its potential implications on our findings.

Notably, only two studies explored racial factors when validating these genomic test tools [27, 31]. Most clinical studies neglected to incorporate the PCa disparities existing among diverse populations. There is a critical need for validating genomic tools in more diverse populations, given the stark disparities in PCa outcomes [74]. This validation is essential for ensuring that precision medicine approaches and risk prediction models are accurate and effective across all racial and ethnic groups, potentially leading to more equitable PCa detection, treatment and outcomes.

## 6 Conclusion

Patients diagnosed with low‐risk or intermediate‐risk PCa are highly heterogeneous, and genomic tests can provide more accurate risk stratification, informing their treatment options and decisions. We reviewed four commercially available genomic test products and identified the clinical evidence supporting the use of these tests to guide PCa treatment decision‐making at the time of diagnosis. Particularly, we systematically evaluated the clinical performance by investigating the reclassification metrics, predictive power and impact on clinical management of these four types of genomic tests. We found that some patients initially stratified by the NCCN guidelines into low and intermediate‐risk categories were reclassified into different risk groups following additional genomic stratification. The highest proportion of reclassification was observed in the GC test studies among PCa patients with intermediate‐risk or FIR features, where the GC test reclassified most patients as low‐risk. The evidence suggests that the genomic test results have significant prognostic value in terms of predicting disease progression, metastasis and cancer‐related death. The genomic test outcomes also influence clinicians' and patients' decisions to select AS and radical treatment, despite other factors such as health literacy and family history also being significant. However, the ProMark test has very limited evidence to support its claims compared to the other three types of genomic tests and may require further investigation.

We reviewed the current health economic evaluation studies and found that they used only minimal clinical evidence compared to our clinical evidence findings. We identified a research gap in conducting a health economic evaluation of using the GC test to guide PCa treatment at the time of diagnosis. The CCP test was evaluated as cost‐saving in the US but not so in Canada. The GPS and the ProMark were evaluated as cost‐effective in the US. However, we observed inconsistencies in the PCa health states, disease progression characteristics and health utilities across these studies, which need further clarification and development. Therefore, the emerging clinical evidence calls for a more comprehensive evaluation of the cost‐effectiveness of using genomic tests to inform PCa treatment decisions, particularly among low‐ and intermediate‐risk groups.

## Author Contributions

**Juntao Lyu:** conceptualization; methodology; formal analysis; project administration; resources; writing – original draft; writing – review and editing; data curation; investigation; supervision; software; visualization; validation. **Fan He:** data curation; investigation; writing – review and editing. **Niall M. Corcoran:** funding acquisition; writing – review and editing; supervision; resources; validation. **Gang Chen:** methodology; supervision; resources; writing – review and editing; investigation; validation; project administration. **Hadi Akbarzadeh Khorshidi:** investigation; supervision; funding acquisition; project administration; resources; writing – review and editing; validation; methodology.

## Funding

This systematic review is part of the Genomically Informed Active Surveillance in Intermediate Risk Prostate Cancer (GenI‐AIRSPACE) project, which is funded by The Advanced Genomics Collaboration (TAGC) and supported by the University of Melbourne, Illumina, and the State Government of Victoria.

## Conflicts of Interest

Juntao Lyu, Gang Chen, Fan He, and Hadi A. Khorshidi are researchers at the Cancer Health Services Research Unit, the University of Melbourne. The unit received funding from the GenI‐AIRSPACE project. Juntao Lyu received salary support from the GenI‐AIRSPACE project. Niall M. Corcoran received grants from TAGC Town Hall—Commercial Innovation Projects to lead the GenI‐AIRSPACE project.

## Supporting information


**Appendix S1:** cam471690‐sup‐0001‐AppendixS1.pdf.

**Table S1:** cam471690‐sup‐0002‐TableS1.docx.

**Table S2:** cam471690‐sup‐0003‐TableS2.xlsx.

**Table S3:** cam471690‐sup‐0004‐TableS3.xlsx.

**Table S4:** cam471690‐sup‐0005‐TableS4.docx.

**Table S5:** cam471690‐sup‐0006‐TableS5.docx.

## Data Availability

The data that supports the findings of this study are available in the Supporting Information of this article.
