# Supplementary material for: Systematic Review of Genomic‐Based Risk Stratification in Localised Prostate Cancer Treatment Optimisation: Clinical Impact and Health Economic Evidence
Source: Cancer Med. 2026 Mar 9;15(3):e71690. doi: 10.1002/cam4.71690 (PMC12971288; doi:10.1002/cam4.71690)
Supplement: Supplementary file 1 — Appendix S1: cam471690‐sup‐0001‐AppendixS1.pdf. [file CAM4-15-e71690-s003.pdf]

Appendix 1: Search Strategies

Step 1: clinical evidence search on Scopus: compare genomic tests as risk stratification with NCCN tests

(((TITLE-ABS-KEY ("decipher genom*")) OR (TITLE-ABS-KEY ("prolaris")) OR (TITLE-ABS-KEY (promark)) OR (TITLE-ABS-KEY ((genome OR genomic OR genomics) W/6 prostate)) OR (TITLE-ABS-KEY ((genome OR genomic OR genomics) W/5 (test* OR assay* OR diagnos* OR classifier* OR stratif* OR analysis OR sequenc*)) OR KEY (genomics)))

AND

((TITLE-ABS-KEY ((prostate OR prostatic) W/3 (cancer* OR tumor* OR tumour* OR neoplas* OR malignan* OR carcinoma OR adenocarcinoma))) OR (TITLE-ABS-KEY (((prostate OR prostatic) W/3 biops*) AND (cancer OR neoplasm*)))))

AND

((TITLE-ABS-KEY ((nccn OR "national comprehensive cancer network") W/5 (test* OR stratif* OR risk OR classif* OR identif* OR score*))) OR (TITLE-ABS-KEY (("transrectal ultrasound" OR trus) W/4 prostate)) OR (TITLE-ABS-KEY (psa OR "prostate specific antigen")) OR (TITLE-ABS-KEY ("rectal exam" OR "rectal examination")))

Number = 985 documents

AND

"low" OR "intermediate" OR "favourable" OR "favorable" OR "localised" OR "localized" OR "early"

Number = 481 documents

Limit to articles or reviews in English:

Number = 424 documents

Step 2: EBM Reviews - Cochrane Central Register of Controlled Trials <September 2024> : compare genomic tests as risk stratification with NCCN tests

1 (prostat* adj3 (cancer or neoplasm*)).mp. 18147

2 ((prostate or prostatic) adj4 (cancer* or tumor* or tumour* or neoplas* or malignan* or carcinoma or adenocarcinoma)).mp. 18699

3 genom*.mp. 8415

4 2 and 3 302

5 (((nccn or "national comprehensive cancer network") adj6 (test* or stratif* or risk or classif* or identif* or score*)) or (("transrectal ultrasound" or trus) adj5 prostate) or psa or "prostate specific antigen" or "rectal exam" or "rectal examination").mp. 11848

Combine

6 4 and 5

Number = 116 documents

Combine

"low" OR "intermediate" OR "favourable" OR "favorable" OR "localised" OR "localized" OR "early"

Number = 69 documents

Step 3: Economic evaluation on Scopus:

(TITLE-ABS-KEY ("economic*" OR "cost" OR "costs" OR "price" OR "market*" OR "pricing"))

AND

(((TITLE-ABS-KEY ("decipher genom*")) OR (TITLE-ABS-KEY ("prolaris")) OR (TITLE-ABS-KEY (promark)) OR (TITLE-ABS-KEY ((genome OR genomic OR genomics) W/6 prostate)) OR (TITLE-ABS-KEY ((genome OR genomic OR genomics) W/5 (test* OR assay* OR diagnos* OR classifier* OR stratif* OR analysis OR sequenc*)) OR KEY (genomics)))

AND

((TITLE-ABS-KEY ((prostate OR prostatic) W/3 (cancer* OR tumor* OR tumour* OR neoplas* OR malignan* OR carcinoma OR adenocarcinoma))) OR (TITLE-ABS-KEY (((prostate OR prostatic) W/3 biops*) AND (cancer OR neoplasm*)))))

Number = 256 documents

AND

"low" OR "intermediate" OR "favourable" OR "favorable" OR "localised" OR "localized" OR "early"

Number = 100 documents

Additional search through Google Scholar and reference lists
