# Supplementary material for: Systematic Review of Genomic‐Based Risk Stratification in Localised Prostate Cancer Treatment Optimisation: Clinical Impact and Health Economic Evidence
Source: Cancer Med. 2026 Mar 9;15(3):e71690. doi: 10.1002/cam4.71690 (PMC12971288; doi:10.1002/cam4.71690)
Supplement: Supplementary file 2 — Table S1: cam471690‐sup‐0002‐TableS1.docx. [file CAM4-15-e71690-s001.docx]

S-Table 1: Data extraction points

| Clinical evidence studies data points | Health economic studies data points |
| --- | --- |
| - Author, title, and year of publication - Genomic test product name - Data source and country of study - Patient cohort characteristics - Study types and follow-up years - Total number of patients - Initial risk stratification groups by NCCN categories - Reclassification by genomic risk stratification - Genomic test predictive power - Impact on treatment - Key conclusions | - Author, title, and year of publication - Country of study and perspectives - Genomic test being evaluated - Clinical evidence sources - Willingness to pay - Costing components (currency and year) - Patient cohort characteristics - Health economic model approaches - Time horizon - Discount rate - Health states (stages of disease) - Health outcomes (effectiveness indicators, lived years, QoL) - Cost-effectiveness outcomes - Sensitivity analysis outcomes - Key conclusions - Limitations |
