# Supplementary material for: Systematic Review of Genomic‐Based Risk Stratification in Localised Prostate Cancer Treatment Optimisation: Clinical Impact and Health Economic Evidence
Source: Cancer Med. 2026 Mar 9;15(3):e71690. doi: 10.1002/cam4.71690 (PMC12971288; doi:10.1002/cam4.71690)
Supplement: Supplementary file 5 — Table S4: cam471690‐sup‐0005‐TableS4.docx. [file CAM4-15-e71690-s006.docx]

**S-Table 4: Summary of the clinical impact evidence studies**

| **Study name** | **Genomic test type** | **Country** | **Patient cohort** | **Follow-up time** | **Study type** | **Genomic risk reclassification*** | **Impact on treatment** | **Predictive power** |
| --- | --- | --- | --- | --- | --- | --- | --- | --- |
| Carbunaru (2023) (35) | GPS | US | NCCN FIR or below, newly diagnosed PCa patients, age ≤ 75 years. | 2-3 weeks | RWD | Reclassified without details | Urologists' preference for RP/RT increased from 14.1% to 29.3% for GPS-tested patients. | Not reported |
| Belkacemi (2023) (36) | GPS | France | NCCN intermediate risk, median age 70 (45–79). | Median 11 months | RWD | 43% (13/30) reclassified | GPS test informed 77% treatment intensification (from RP/RT to RP/RT + ADT) and 3% treatment de-escalation. | Significant correlation between GPS and the percentage of Gleason Grade 4 or higher patterns in surgical samples. |
| Seiden (2022) (27) | GPS | US | Low grade PCa (Gleason 3+3 or 3+4), black race, median age 66. | 17 months | RWD | 38% (21/55) reclassified | 67% (12/18) undertook radical treatment after being classified to higher risks | Not reported |
| Murphy (2021) (33) | GPS | US | NCCN FIR or below, newly diagnosed PCa patients, age ≤ 75 years. | 6-7 weeks | RCT | 32% (20/62) reclassified | AS utilisation decreased from 88% (control group) to 74% (GPS group) | Not reported |
| Greenland (2020) (37) | GPS | US | NCCN intermediate risk or below | Median 1.6 years | RWD | 12% (56/480) reclassified | Not reported | GPS test was associated with moderate or severe stromal reaction (p < .001), chronic inflammation (p < .001), and the presence of nuclear polarisation. |
| Lin (2020) (38) | GPS | US, Canada | NCCN intermediate risk or below, median age 63 | Median 4.6 years | RWD | Not reported | Not reported | No association observed between GPS and subsequent biopsy upgrade. |
| Gaffney (2019) (39) | GPS | US | NCCN intermediate risk or below, median age 65 | 3 years | RWD | 17% (17/103) reclassified | 75% (9/12) opted for AS after GPS reclassified from intermediate to low risk. | Not reported |
| Nyame (2018) (40) | GPS | US | NCCN low risk or below, median age 63 | Median 1.5 years | RWD | 7% (13/183) reclassified |  |  |
| Eure (2017) (41) | GPS | US | NCCN intermediate risk or below, median age 65 | 1 year | RWD | 8% (16/190) reclassified | AS utilisation increased from 40% to 62% after GPS test | Not reported |
| Albala (2016) (42) | GPS | US | NCCN FIR or below. median age 60 (42-80), 83% stage T1c disease. | 17 months | RWD | 18% (10/57) reclassified | AS utilisation increased by 21% after GPS test | Not reported |
| Badani (2015) (29) | GPS | US, Canada | NCCN intermediate risk or below, newly diagnosed PCa patients, median age 64. | Unclear | RWD | 15% (18/123) reclassified | AS utilisation increased from 41% to 51% after GPS test | Not reported |
| Dall’Era (2015) (43) | GPS | US | NCCN intermediate risk or below, newly diagnosed PCa patients, median age 56. | 9 months | RWD | Not reported | AS utilisation increased from 50% to 61% after GPS test | Not reported |
| Spratt (2023) (34) | GC | US | NCCN intermediate risk, median age 70 | Median 12.8 years | RCT | 90% (194/215) reclassified | Higher GC scores benefit from treatment intensiﬁcation, potentially via pelvic nodal dissection, pelvic nodal irradiation, and/or the addition of chemohormonal agents. | GC score 0.1 unit increase predicting disease progression ((sHR = 1.12, 95% CI: 1.002-1.26, p=0.04); metastasis (sHR = 1.28, 95% CI: 1.06-1.55, p=0.01); and PCa-specific mortality ( sHR = 1.45, 95% CI: 1.20-1.76, p<0.001). |
| Zaorsky (2023) (30) | GC | US | PCa patients age > 40 | Unclear | RWD | 63% (1818/2885) reclassified | AS utilisation increased from 26.9% to 57.4% among the low risk groups, from 12.6% to 29.1% among the FIR patients after GC test. | Not reported |
| Press (2022) (44) | GC | US | PCa biopy GG1 & GG2, median age 68 | Median 4.1 years | RWD | 10.3% (15/133) reclassified | Not reported | GC score 0.1 unit increase associated with biopsy upgrading among patients with GG1 (OR = 1.29 per 0.10 unit; p = 0.047) but not among those with GG2 disease. |
| Vince (2022) (45) | GC | US | Median PSA level was 5.9 ng/ml, 83% of patients had cT1 or T2a disease at diagnosis, median age 66. | 15-25 months | RWD | 14% (16/116) reclassified | Patients with high-risk GC scores had a significantly greater likelihood of undergoing definitive treatment (HR 2.51, 95% CI 1.52–4.13; p <0.001) and spent significantly less time on AS (median 13.6 months, IQR 3.5–24.9) than men with GC low/intermediate risk scores (median 33.0 months; p<0.001). | Not reported |
| Herlemann (2020) (46) | GC | US | NCCN FIR, median age 61 | Median 2.8 years | RWD | 87% (191/220) reclassified | Not reported | GC was a significant predictor of adverse pathology with an odds ratio of 1.34 per 0.1 unit increase (p = 0.002). |
| Xu (2019) (31) | GC | US | NCCN intermediate risk and above, median age 69 | Unclear | RCT | 100% (23/23) reclassified | Not reported | GC score 0.1 unit increase associated with 40% increase in the odds of PSMA-avid lymph node involvement, not signiﬁcantly associated with PSMA-avid osseous metastases. |
| Hutten (2024) (47) | CCP | US | NCCN intermediate risk, median age 67 | Median 3.2 years | RWD | Not reported | Not reported | CCP score (1 unit increase) was prognostic for metastasis (HR = 2.32; CI, 1.17- 4.59; P = 0.02), and the AUC for a 3-year risk of metastasis on the basis of CCP was 0.736. |
| Tward (2021) (48) | CCP | US | NCCN intermediate risk and above | 5-10 years |  | 50% (261/525) reclassified | Not reported | CCP score 1 unit increase corresponded to 4 times increase in risk of metastasis (HR = 3.75; 95% CI, 2.71-5.2, p <0.0001). |
| Rayford (2018) (28) | CCP | US | African American and Caucasian American PCa patients, PSA range from 3.6-8.8 ng/mL, median age 66 | Unclear | RWD | 395 (55/140) reclassified | Not reported |  |
| Shore (2016) (49) | CCP | US | PCa patients, median age 66 | >3 months | RWD | Not reported | 24.2% (101/417) changed from noninterventional to interventional, and 14.2% (112/789) changed from interventional to noninterventional treatment options after the CCP test. | Not reported |
| Cuzick (2015) (50) | CCP | UK | Localised PCa patients, clinical stage 1-4, median age 71 | Median 9.5 years | RWD | Not reported | Not reported | CCP score 1 unit increase corresponded to death from PCa (HR = 2.92; CI, 2.38- 3.57; P <0.0001). |
| Cuzick (2012) (51) | CCP | UK | Localised PCa patients conservatively managed, age < 76 | Median 11.8 years | RWD | Not reported | Not reported | CCP score 1 unit increase corresponded to death from PCa (HR = 2.02; CI, 1.62- 2.53; P <0.0001). |
| Cuzick (2011) (52) | CCP | UK | Localised PCa patients conservatively managed, age < 76 | Median 9.8 years | RWD | Not reported | Not reported | CCP score 1 unit increase corresponded to death from PCa (HR = 2.08; CI, 1.76- 2.46; P <0.0001). |
| Blume-Jensen (2015) (32) | ProMark | US | PCa patients with Gleason core 3+3 & 3+4, mean age 60 | Unclear | RWD | 58% (105/182) reclassified | Not reported | The predictive value for nonfavourable pathology was 76.9% at ProMark score > 0.8 across all risk groups. |

**The overall genomic reclassification percentages are calculated based on n/N in each study, n = the number of patients being reclassified to lower or higher risk groups, and N = the total number of patients with intermediate or below risk of PCa.*

*PCa: Prostate cancer. GPS: Genomic Prostate Score, also called Oncotype DX; GC: Genomic Classifier, also called Decipher Prostate test; CCP: Cell Cycle Progression, also called Prolaris. RWD: Real world data; RCT: Randomised clinical trials. RP: Radical prostatectomy; RT: Radiotherapy; AS: Active surveillance; NCCN: National Comprehensive Cancer Network.*
