# Supplementary material for: Systematic Review of Genomic‐Based Risk Stratification in Localised Prostate Cancer Treatment Optimisation: Clinical Impact and Health Economic Evidence
Source: Cancer Med. 2026 Mar 9;15(3):e71690. doi: 10.1002/cam4.71690 (PMC12971288; doi:10.1002/cam4.71690)
Supplement: Supplementary file 6 — Table S5: cam471690‐sup‐0006‐TableS5.docx. [file CAM4-15-e71690-s005.docx]

**S-Table 5: Genomic Risk Reclassification Metrics**

| **Initial NCCN risk group** | **Number of patients** | **Genomic test** | **Reclassified risk group after genomic test** | | | **Study name** |
| --- | --- | --- | --- | --- | --- | --- |
|  |  |  | **Low risk** | **Intermediate risk** | **High risk** |  |
| Intermediate risk | N = 296 | GPS | n = 33 (11.1%) | -# | n = 8 (2.7%) | Greenland (2020) |
|  | N = 58 | GPS | n = 12 (20.7%) | - | n = 1 (1.7%) | Gaffney (2019) |
|  | N = 79 | GPS | n = 8 (10.1%) | - | n = 0 (0.0%) | Eure (2017) |
|  | N = 215 | GC | n = 174 (81.0%) | - | n = 20 (9.0%) | Spratt (2023) |
|  | N = 23 | GC | n = 16 (69.6%) | - | n = 7 (30.4%) | Xu (2019) |
|  | N = 158 | GC | n = 66 (41.8%) | - | n = 39 (24.7%) | Zaorsky (2023 |
|  | N = 85 | CCP | n = 28 (32.9%) | - | n = 15 (17.6%) | Rayford (2018) |
|  | N = 88 | ProMark | n = 12 (13.6%) | - | n = 26 (29.5%) | Blume-Jensen (2015) |
| FIR | N = 28 | GPS | n = 4 (14.3%) | - | n = 6 (21.4%) | Murphy (2021) |
|  | N = 18 | GPS | n = 0 (0.0%) | - | n = 7 (38.9%) | Belkacemi (2023) |
|  | N = 31 | GPS | n = 11 (35.5%) | - | n = 3 (9.7%) | Seiden (2022) |
|  | N = 29 | GPS | n = 0 (0.0%) | - | n = 0 (0.0%) | Albala (2016) |
|  | N = 52 | GPS | n = 15 (28.9%) | - | n = 0 (0.0%) | Badani (2015) |
|  | N = 769 | GC | n = 323 (42%) | - | n = 219 (28.5%) | Zaorsky (2023) |
|  | N = 50 | GC | n = 11 (22.0%) | - | n = 2 (4.0%) | Vince (2022) |
|  | N = 220 | GC | n = 174 (79.1%) | - | n = 17 (7.7%) | Herlemann (2020) |
|  | N = 171 | CCP | n = 3 (1.8%) | - | n = 0 (0.0%) | Tward (2021) |
| UIR | N = 12 | GPS | n = 2 (16.7%)## | - | n = 4 (33.3%) | Belkacemi (2023) |
|  | N = 952 | GC | n = 318 (33.4%) | - | n = 393 (41.3%) | Zaorsky (2023) |
|  | N = 354 | CCP | n = 258 (72.9%) | - | n = 0 (0.0%) | Tward (2021) |
| Low risk | N = 34 | GPS | - | n = 10 (29.4%) | n = 0 (0.0%) | Murphy (2021) |
|  | N = 24 | GPS | - | n = 7 (29.2%) | n = 0 (0.0%) | Seiden (2022) |
|  | N = 184 | GPS | - | n = 15 (8.2%) | n = 0 (0.0%) | Greenland (2020) |
|  | N = 45 | GPS | - | n = 4 (8.9%) | n = 0 (0.0%) | Gaffney (2019) |
|  | N = 183 | GPS | - | n = 13 (7.1%) | n = 0 (0.0%) | Nyame (2018) |
|  | N= 111 | GPS | - | n = 8 (7.2%) | n = 0 (0.0%) | Eure (2017) |
|  | N = 28 | GPS | - | n = 10 (35.7%) | n = 0 (0.0%) | Albala (2016) |
|  | N = 71 | GPS | - | n = 3 (4.2%) | n = 0 (0.0%) | Badani (2015) |
|  | N = 1006 | GC | - | n = 287 (28.5%) | n = 173 (17.2%) | Zaorsky (2023) |
|  | N = 66 | GC | - | n = 0 (0.0%) | n = 3 (4.5%) | Vince (2022) |
|  | N = 55 | CCP | - | n = 11 (20.0%) | n = 1 (1.8%) | Rayford (2018) |
|  | N = 94 | ProMark | - | n = 59 (62.8%) | n = 8 (8.5%) | Blume-Jensen (2015) |

*# The reclassification metrics only show the number of patients being reclassified into lower or higher risk groups. The dashed line marked the remaining patients with the same risk classification who are not being reclassified.*

*##The two patients were reclassified from unfavourable to favourable.*

*GPS: Genomic Prostate Score, also called Oncotype DX; GC: Genomic Classifier, also called Decipher Prostate test; CCP: Cell Cycle Progression, also called Prolaris; FIR: Favourable intermediate risk; UIR: unfavourable intermediate risk.*
